# Neuroprotective effects of hypothermia and levetiracetam after hypoxia-ischemia in the neonatal mouse brain

**DOI:** 10.1186/2194-7791-1-S1-A7

**Published:** 2014-09-11

**Authors:** Katja Straßer, Daniela Brait, Laura Lückemann, Barbara Simone Reinboth, Josephine Herz, Ivo Bendix, Ursula Felderhoff-Müser

**Affiliations:** Department of Pediatrics 1, Neonatology, University Hospital Essen, Essen, Germany

## Aims

Hypoxic-ischemic injury (HI) to the developing brain remains a major cause of morbidity. Hypothermia is currently the only established neuroprotective treatment available for term borns with hypoxic-ischemic encephalopathy, saving one in eight infants from developing severe neurological deficits. Therefore, additional treatments with clinically applicable drugs are indispensable. Furthermore, the pathophysiological mechanisms of hypothermia-induced recovery are not clearly understood. This study examines a potential additive neuroprotective effect of hypothermia combined with levetiracetam in neonatal mouse HI.

## Methods

9-days-old C57BL/6-mice were subjected either to a sham-operation or to HI (modified Rice-Vannucci-model). After HI, the pups were randomized into six groups: 1) no treatment, 2) hypothermia (whole body-cooling, 4 hours, 32°C), 3) high-dose levetiracetam intraperitoneal (70 mg/kg body weight), 4) hypothermia combined with high-dose levetiracetam intraperitoneal, 5) low-dose levetiracetam intraperitoneal (7 mg/kg body weight), 6) hypothermia combined with low-dose levetiracetam intraperitoneal. Parameters of apoptosis (cleaved Caspase-3, TUNEL) and myelination (myelin basic protein) were analyzed 24 hours after HI by protein analysis and immunhistochemistry. From P28 to P60, cognitive and sensorimotor function was assessed via different tests.

## Results

Hypothermia only and combined with low-dose levetiracetam was associated with a decrease of apoptosis and an increase of myelinated cells, but without additive effects. Intraperitoneal treatment with high-dose levetiracetam caused an increase of apoptotic factors. Behavioural testing demonstrated improved cognitive and sensorimotor outcome after treatment with hypothermia.

## Conclusion

Whole-body cooling provides neuroprotection in the neonatal mouse brain by reducing apoptosis and preservation of myelination. However, treatment with levetiracetam after hypoxic-ischemic injury has no additive effects.

